# Similarities and Differences of Multiple Epiphyseal Dysplasias: Genetic Features and Natural Course in 22 Patients

**DOI:** 10.3390/genes17040463

**Published:** 2026-04-15

**Authors:** Hasan Emir Taner, Dilek Uludağ Alkaya, Ayşe Kalyoncu Uçar, Ali Şeker, Tuncay Centel, Timur Yıldırım, Nilay Güneş, Beyhan Tüysüz

**Affiliations:** 1 Department of Pediatric Genetics, Cerrahpaşa Medical Faculty, Istanbul University-Cerrahpaşa, 34098 Istanbul, Turkey; hasanemirtaner@gmail.com (H.E.T.); dilek.uludagalkaya@iuc.edu.tr (D.U.A.); nilay.gunes@iuc.edu.tr (N.G.); 2 Department of Radiology, Cerrahpaşa Medical Faculty, Istanbul University-Cerrahpaşa, 34098 Istanbul, Turkey; ayse.kalyoncuucar@iuc.edu.tr; 3 Department of Orthopedics and Traumatology, Cerrahpaşa Medical Faculty, Istanbul University-Cerrahpaşa, 34098 Istanbul, Turkey; ali.seker@iuc.edu.tr; 4 Independent Researcher, 34158 Istanbul, Turkey; dr@tuncaycentel.com (T.C.); drtimuryildirim@gmail.com (T.Y.); 5 Department of Pediatrics, Istanbul Atlas University, 34408 Istanbul, Turkey

**Keywords:** multiple epiphyseal dysplasia, *COMP*, *MATN3*, *COL9A2*, *COL9A3*, *CANT1*, *SLC26A2*, phenotype variability

## Abstract

**Background/Objectives:** Multiple epiphyseal dysplasia (MED) is a clinically and genetically heterogeneous group of disorders characterized by a waddling gait, joint pain, and early-onset osteoarthritis. The aim of this study was to compare the genetic characteristics and long-term clinical follow-up findings of 22 patients with MED from 17 unrelated families. **Methods:** Molecular diagnosis was performed using clinical exome analysis and exome sequencing. Seventeen children were followed for a median of 5.5 years. **Results:** Eighteen disease-related variants were identified: 47% in *COMP*, 11.8% each in *COL9A2* and *COL9A3* in a monoallelic state, 17.6% in *SLC26A2*, and 11.8% each in *MATN3* and *CANT1* in a biallelic state. Some *COMP* mutations previously identified in pseudoachondroplasia, an allelic disorder of MED1, were shown in our study to exhibit a typical MED1 or intermediate phenotype. In contrast, it was confirmed that certain mutations in *SLC26A2* lead to MED4 phenotype. Furthermore, it has been observed that biallelic variants in *MATN3* may be associated with the MED5 phenotype. In patients with MED2 and MED3, the knee joint is affected, while in other types, the hip joint is predominantly affected. In 15 children followed until ages 11–18, height decreased slightly as they grew older but remained normal or at the lower limit, and slow progression was observed in the waddling gait and joint pain, except in the intermediate form. **Conclusions:** This study reveals the frequency of disease-related variants, including seven novel ones, in genes leading to MED1–5 and 7 phenotypes, and expands the spectrum of genetic and clinical phenotypes.

## 1. Introduction

Multiple epiphyseal dysplasia (MED) is a group of disorders characterized by waddling gait, fatigue, and joint pain after exercise in early childhood. Early-onset osteoarthritis may develop in the hip and knee joints, sometimes requiring joint replacement in the third or fourth decade of life [1,2,3]. The clinical diagnosis is made when more than one epiphysis of a long bone is symmetrically irregular, small, and/or fragmented on skeletal radiographs [3,4,5]. Some patients may show negligible metaphyseal or vertebral endplate irregularities. Although the prevalence of autosomal dominant forms is reported to be 1 in 10,000–20,000, it is estimated to be higher [1].

Multiple epiphyseal dysplasia is genetically heterogeneous; monoallelic pathogenic variants in *COMP* (MED1; MIM#132400), *COL9A2* (MED2; MIM#600204), *COL9A3* (MED3; MIM#600969), *MATN3* (MED5; MIM#607078), and *COL9A1* (MED6; MIM#614135), as well as biallelic variants in *SLC26A2* (MED4; MIM#226900) and *CANT1* (MED7; MIM#617719), cause MED [6]. The first gene identified in MED was *COMP*, which accounts for 50% of cases, while *COL9A1*, *COL9A2*, and *COL9A3* are responsible for 10% of cases, and *MATN3* for 20%. In 20% of cases, the pathogenic variant cannot be identified [7,8]. The genes responsible for these seven MED phenotypes encode key proteins of the extracellular cartilage matrix, or enzymes and sulphate carrier proteins involved in cartilage metabolism. Cartilage oligomeric matrix protein (COMP) is a large extracellular glycoprotein that binds to matrilin-3 (MATN3) and collagen types II and IX and plays a role in calcium binding and calcium-induced protein folding in growth plate chondrocytes. SLC26A2 enables the sulphation of key structural components of the extracellular matrix, including proteoglycans, aggrecans, and collagen-IX proteins in cartilage [2]. CANT1 is an enzyme involved in the formation of proteoglycans [9]. Defects in these genes disrupt the extracellular matrix composition and molecular interactions of cartilage, ultimately leading to the destruction of the joint cartilage [10,11,12].

Clinical findings and the natural course are quite heterogeneous among the different types; furthermore, clinical findings may overlap with allelic disorders, bilateral Legg–Calvé–Perthes disease, Stickler syndromes, and spondyloepiphyseal dysplasia [12,13]. The genes that cause MED lead to allelic disorders, which are characterized by severe short stature. Monoallelic mutations in *COMP* cause the allelic disorder pseudoachondroplasia (PSACH; MIM#77170), characterized by severe short stature, short limbs, and spondyloepimetaphyseal dysplasia (SEMD). Patients may present with a spectrum of phenotypes between the two disorders, ranging from MED1 to PSACH [1,7,14]. Furthermore, monoallelic mutations in *COL9A1-COL9A3* lead to MED phenotypes, while biallelic variants cause Stickler syndrome, characterized by epiphyseal dysplasia, hearing and vision problems. Similarly, monoallelic mutations in *MATN3* cause MED5, while biallelic mutations cause spondyloepimetaphyseal dysplasia (SEMD) Borochowitz–Cormier-Daire type (MIM#608728), characterized by severe short stature with short limbs [15]. Biallelic mutations in *SLC26A2*, in addition to MED4, cause diastrophic dysplasia (MIM#222600), characterized by severe short-limbed stature, hypertrophic auricular cartilage, cleft palate, and talipes equinovarus [2]. In addition, biallelic mutations in *CANT1* are associated with Desbuquois dysplasia (MIM#251450), which includes short-limb dwarfism, and joint dislocation; only a few cases have been reported in which they cause MED7 [9].

Clinical phenotyping can be difficult because the same genes give rise to allelic disorders and intermediate phenotypes. The aim of this study is to investigate genotype–phenotype correlations and the natural course of patients with disease-related variants in the genes associated with MED, and to compare common and distinctive clinical findings among the different types.

## 2. Materials and Methods

The study included 22 patients from 17 unrelated families, whose clinical findings were consistent with MED and who were followed at a single center. Patients with variants in the MED genes causing the disease, but whose clinical findings were consistent with an allelic disorder rather than MED, were excluded from the study, while five patients with an intermediate phenotype were included.

Clinical characteristics, including anthropometric measurements (weight, height, head circumference, arm span, sitting height, and hand length), were recorded at the first admission and at annual follow-up examinations. The standard deviation score (SDS) for all anthropometric measurements was calculated using a national pediatric calculator based on national standards (https://www.ceddcozum.com, accessed on 3 April 2026). Detailed histories were obtained from the patients, their parents, siblings, and other affected relatives with a similar history. X-ray examinations of the skeleton were performed at enrolment and at annual follow-up visits. Biochemical blood analysis, urinalysis, eye examination, hearing test, echocardiography, and abdominal ultrasonography were performed at enrolment and at follow-up visits.

Genomic DNA was extracted from the peripheral blood leukocytes of patients and parents using the salting-out DNA isolation method. Clinical exome analysis was performed in 17 families. In cases where the clinical exome did not yield pathogenic or likely pathogenic variants, exome sequencing analysis was performed. All variants identified were confirmed by Sanger sequencing in the probands and their parents.

Rare variants were selected based on a minor allele frequency of less than 1% in public databases, a minimum quality score of 30, and a read depth of at least 10×. Bioinformatics tools (PolyPhen2, SIFT, MutationTaster, DANN, SpliceAI) and electronic databases (dbSNP, ExAC, 1000G, ClinVar, Varsome, HGMD Professional Version) were used to assess the pathogenicity of the variants. The pathogenicity of the variants was interpreted according to American College of Medical Genetics and Genomics (ACMG) guidelines. Selected variants, including pathogenic, likely pathogenic and variants of uncertain significance (VUS), were confirmed by Sanger sequencing using an ABI PRISM 3500 genetic analyser (Applied Biosystems, Foster City, CA, USA). After all variants were confirmed in the proband, segregation analysis was performed on the mother, father, and all siblings.

## 3. Results

The study cohort comprised 22 patients (13 males, 9 females; four parents) from 17 unrelated families. Eighteen children were followed for a median duration of 5.5 years (range 1–9.5 years). Among the 22 patients, 18 disease-related variants were identified, seven of which were novel (Table 1). Monoallelic pathogenic variants in *COMP* were found in eight families (47%), and in *COL9A2* and *COL9A3* in one family each (11.8%). Biallelic variants were present in *SLC26A2* (17.6%) in three families, and in *MATN3* (11.8%) and *CANT1* (11.8%) in two families each. The detailed clinical and radiological findings of 22 patients from 17 families with MED are summarized in Supplementary Tables S1 and S2.

### 3.1. Molecular and Clinical Features of Patients with MED1

**Figure 1:**
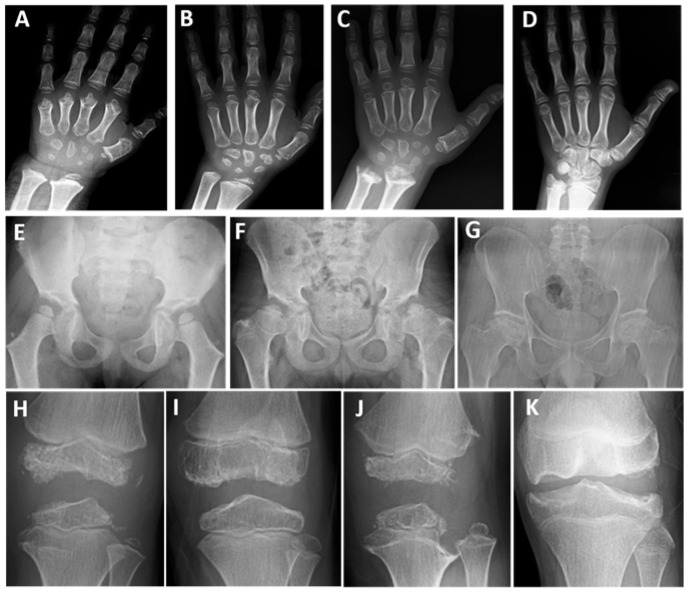
Patients with MED1. P1 at 5 years of age (**A**), P8 at 9 years of age (**B**), and P7 at 8 and 14.5 years of age (**C**,**D**). Note the ragged, small, and irregular carpal bones (**A**–**C**) and the short metacarpal bones (**A**,**C**) in early childhood; in adolescence, only slightly small and irregular carpal bones are present (**D**). P7 at 5.7 years of age (**E**), P8 at 12 years of age (**F**), and P10 at 39 years of age (**G**). All patients had an irregular acetabular contour or a dysplastic acetabulum; note the small, round femoral heads (mini-epiphysis) and coxa valga (**E**). The femoral heads are flat and irregular, and the femoral necks are short and broad; coxa vara developed with age (**F**,**G**). P8 at 6.5 years of age (**H**), P9 at 8.5 years of age (**I**), P7 at 8 years of age (**J**), and P5 at 13 years of age (**K**). The knee epiphyses were irregular and flattened (**H**–**K**), with the ‘glacier crevice’ sign (**H**,**J**). Slight metaphyseal irregularities of the tubular bones were also noted in P7 (**C**,**J**).

In ten patients diagnosed with MED1 (eight children and two parents), two novel and eight pathogenic or likely pathogenic monoallelic variants were present in *COMP* (Table 1). In eight children, the mean age of onset of symptoms such as waddling, fatigue, or joint pain was 4.5 years (Table 2 and Table S1). The mean height at presentation was approximately −1.2 SDS. Seven patients were between 10 and 15 years old at the last examination, with a mean height of −1.5 SDS (Table 2). Final height was −2 SDS and 0.6 SDS in two parents (Table S1). Limited elbow joint was present in eight patients, genu varum in one, and genu valgum deformity in four. Pes planus was observed in six patients. In eight of the 10 patients, the hip joint was more severely affected. In children aged between 10 and 15 at the last examination, progressive waddling gait and fatigue developed during walking; they experienced significant difficulty in climbing stairs and presented with increased hip and/or knee joint pain. One patient’s father underwent surgery for early-stage hip osteoarthritis. In childhood, radiographs showed small, ragged, and irregular carpal bones and short metacarpal bones (Figure 1A–C), while in adolescence only slightly irregular carpal bones were observed (Figure 1D). Typical small, round epiphyses were present at the femoral heads at 5–7 years of age, whereas after 8 years of age, they became flattened and irregular, and the femoral necks shortened and widened (Figure 1E–G). All patients had varying degrees of irregular, flat, or small epiphyses at the knees; small epiphyses with lateral thinning, and additional ossification centers with the ‘glacier crevice sign’ were observed in some patients before puberty (Figure 1H–K). Slight metaphyseal irregularities were present in two patients with the MED1-PSACH intermediate phenotype (Figure 1C,J).

### 3.2. Molecular and Clinical Features of Patients with MED2 and MED3

**Figure 2:**
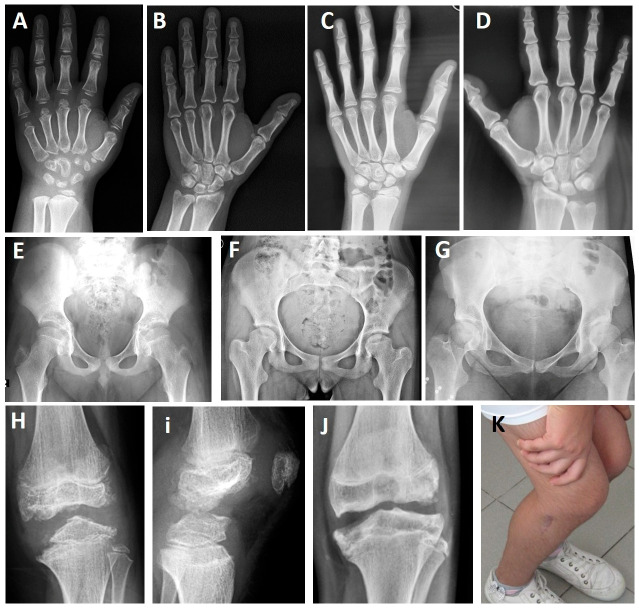
Patients with MED2 and MED3. P11 at age 10 years (**A**,**E**,**H**,**I**) and P12 at age 45 years (**B**) with MED2. P13 at age 14 years (**C**,**F**,**J**) and P14 at age 49 years (**D**,**G**,**K**) with MED3. Small and irregular carpal bones are seen in childhood (**A**,**C**), while slight irregularities of the carpal bones are observed in adulthood (**B**,**D**) on hand radiographs. Note the normal features (**E**) in the patient with MED2 on the pelvic radiograph, while a flat femoral head (**F**) and a small femoral head with a short femoral neck are seen in MED3 (**G**). Irregular knee epiphyses are observed in patients with MED2 and MED3 (**H**–**J**). Knee limitation observed in P13 (**K**).

P11 and his father (P12) had a monoallelic VUS (c.186+6T>G) in *COL9A2*. P13 and his mother (P14) had a monoallelic likely pathogenic variant in *COL9A3* (Table 1). P11 and P13 began to experience knee pain at ages 4 and 5, respectively, and later developed increased pain during long walks and difficulty climbing stairs after age 10. P13 had a history of corrective surgery for genu varum deformity, and his affected mother underwent knee replacement surgery at age 40 due to osteoarthritis. Both children had small and irregular carpal bones on skeletal radiographs (Figure 2A,C) at ages 10 and 14, respectively, while their affected parents had only mild irregularities in the carpal bones (Figure 2B,D). Hip X-rays were normal in P11 and his father (P12) (Figure 2E), while 11-year-old P13 had a slightly flattened femoral head (Figure 2F), and his mother (P14) had a short femoral neck and a slightly smaller femoral head (Figure 2G). The knee epiphyses of P11 (Figure 2H,I), P13 (Figure 2J), and P14 (Figure 2K) were quite irregular and destroyed.

### 3.3. Molecular and Clinical Features of Patients with MED4

**Figure 3:**
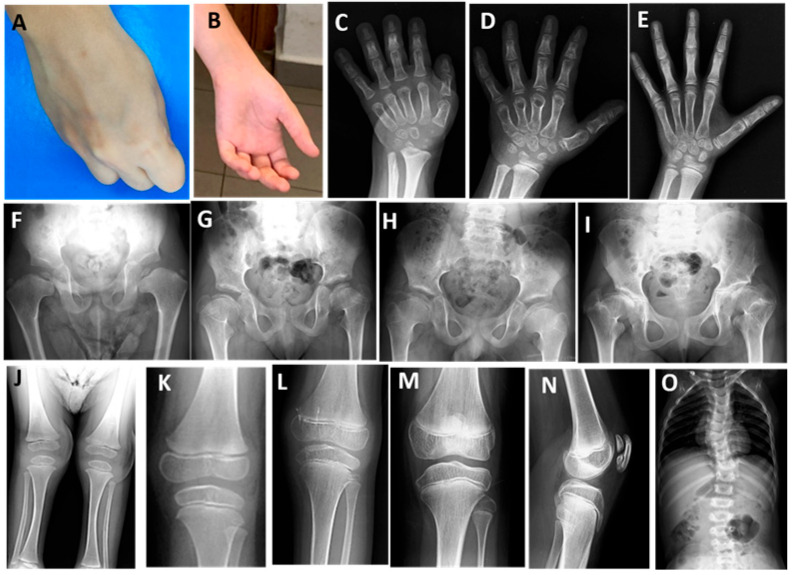
Patients with MED4. P15 at 11 years (**A**,**I**,**M**,**N**) and 5 years (**E**), P16 at 7 years (**B**,**H**,**L**), P17 at 3 years (**D**) and 5 years (**G**,**K**), and P18 at 2.5 years (**C**,**F**,**J**,**O**). Note the flexion contracture of the fingers (**A**,**B**), advanced bone age (**C**–**E**), metaphyseal irregularity (**C**), and shortening of the metacarpal bones (**D**). Coxa vara with broad, short femoral necks and small femoral epiphyses (**F**), flat and crescent-shaped femoral epiphyses with broad and short femoral necks (**G**,**H**,**I**) and protruding trochanter were revealed on pelvic radiographs (**I**). Note the short lower limb (**J**), broad metaphyses and flattened knee epiphyses (**K**–**M**), the classic appearance of a ‘double-layered patella’ on the lateral radiograph of the knee (**N**), and scoliosis (**O**).

Biallelic variants, including one compound heterozygous variant, were detected in *SLC26A2* in three families; two were previously reported as VUS, one was novel, and one was a recurrent pathogenic variant (Table 1). Four patients with MED4 (P15–P18) experienced waddling gait, fatigue after exercise, and contractures of the finger joints; they were unable to make a fist due to flexion contractures (Table S2, Figure 3A,B). Bilaterally operated clubfoot, broad, short hands and feet, and mildly short limbs were noted in P18. During follow-up, the waddling gait did not worsen with prolonged walking and joint pain began in late childhood. Of the two patients who reached age 11, one had a height of −1.5 SDS and the other −1.9 SDS. Radiographs of the hands of all patients showed advanced bone age, flat carpal bones, and shortened metacarpal and metatarsal bones (Figure 3C–E). X-ray images of the patients at different ages showed flat and crescent-shaped femoral epiphyses, broad and short femoral necks (Figure 3F–I), slightly flattened knee epiphyses (Figure 3J–M) and one developed a double-layered patella (Figure 3N). Mild limb shortening (Figure 3J) and scoliosis (Figure 3O) were also present in P18 with intermediate phenotype.

### 3.4. Molecular and Clinical Features of Patients with MED5

**Figure 4:**
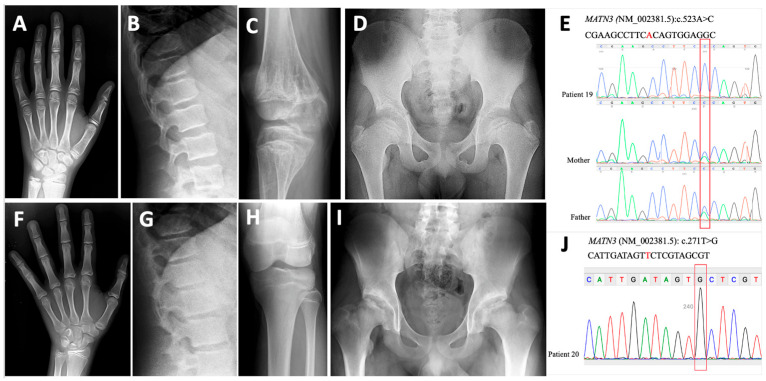
Patients with MED5. P19 at the age of 14 years. Note mild carpal irregularity (**A**) and platyspondyly (**B**), severe irregularities in the knee epiphyses with metaphyseal striae (**C**), flattening of the femoral head, and coxa vara (**D**). Sanger sequencing of the VUS identified in P19 and segregation in the family (**E**). Radiographs of P20 with MED5 at the age of 14 years showed mild carpal irregularity (**F**) and platyspondyly (**G**), a normal knee joint (**H**), and acetabular irregularity with destroyed femoral heads (**I**). Sanger sequencing of the VUS identified in P20 by exome sequencing (**J**).

In two patients (P19 and P20) with biallelic VUSs (c.271T>G and c.523A>C) in *MATN3*, knee and hip pain began in prepuberty, respectively. The height of P19 was −3 SDS, while that of P20 was +2 SDS at the age of 14 years. Skeletal screening of P19 and P20 revealed mild carpal irregularities (Figure 4A,F). P19 had severe irregularities of the knee epiphyses with metaphyseal striations (Figure 4C), whereas normal knee epiphyses were present in P20 (Figure 4H). P20 had short femoral necks and destroyed femoral heads (Figure 4I), while P19 had only slight flattening of the femoral head (Figure 4D). The clinical findings of these two patients were consistent with MED, with no vertebral or metaphyseal involvement (Figure 4A–C,F–H). Exome sequencing was performed on both children after clinical exome analysis, and no additional disease-causing variants were found. In the segregation analysis, the healthy parents of P19 were heterozygous for the same variant (Figure 4E). The VUS in P20 was confirmed by Sanger sequencing (Figure 4J), but segregation analysis could not be performed in the healthy parents.

### 3.5. Molecular and Clinical Features of Patients with MED7

**Figure 5:**
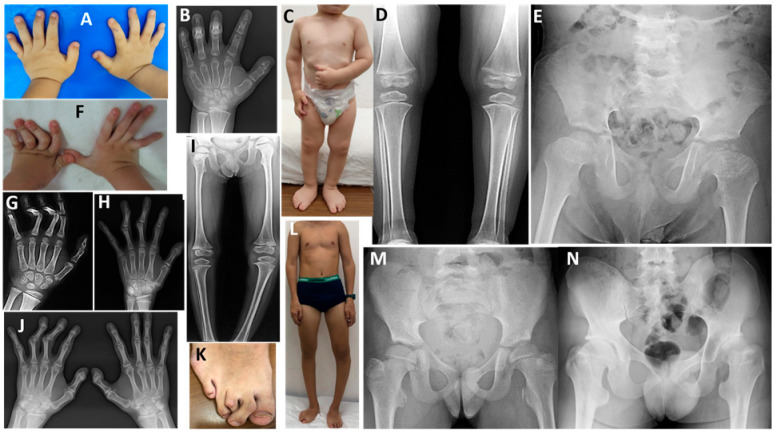
Patients with MED7. P21 at the age of 3 years. Note mild flexion contracture of the distal interphalangeal joints (**A**), advanced carpal bones and mild metacarpal shortening (**B**), broad feet with pes planus (**C**), small epiphyses at the knees (**D**), and horizontal acetabula, flat and irregularly shaped capital femoral epiphyses, broad femoral neck, and mild ‘Swedish key’ appearance of the proximal femur (**E**). P22 with MED7 at 3.5 years (**F**,**G**,**I**,**M**), 10 years (**H**), 14 years (**L**), and 17 years (**J**,**K**,**N**) revealed severe flexion contracture of the 3rd and 4th fingers at the left interphalangeal joints (**F**), phalangeal dislocation and advanced carpal bones (**G**,**H**,**J**), flexion contracture of the 2nd and 3rd toes on the right (**K**), genu varum deformities of the left limb (**I**,**L**), ‘Swedish key’ appearance of the proximal femur (**M**,**N**), and coxa valga (**N**).

Biallelic c.375G>C and c.-342+1G>A reported variants of *CANT1* were identified in two children aged 1.5 and 3 years, who presented with short stature (−2.4 and −3.2 SDS), flexion contractures of the fingers and pes planus, respectively. Both had round faces, mild midface hypoplasia, mild to moderate short stature, and flexion contractures of fingers, but no short limbs (Figure 5A,C,F,K,L). Radiological examination of the skeleton of the two patients revealed osteopenia, advanced carpal bone age, mild metacarpal shortness, normal phalangeal lengths, phalangeal dislocation, flat acetabula, hypoplastic and irregular capital femoral epiphyses, coxa valga, short femoral necks, and a ‘Swedish key’ appearance of the proximal femur (Figure 5B,D,E,G–I,J,M,N). The patient diagnosed at 1.5 years of age was followed up until 18 years; at this age, his height was −3.6 SDS. He underwent foot and knee surgery due to club feet and genu valgus; however, he had no joint pain, no difficulty in walking or climbing stairs, and his school performance was very good.

When comparing the clinical findings of different MED types (Table 2), we observed that the initial symptoms were mostly waddling gait and joint pain, beginning in early childhood. Interestingly, clubfoot in MED4 and MED7 may suggest an initial diagnosis in infancy. Two patients with *COMP* variants, one with an *SLC26A2* variant, and two with a *CANT1* variant showed an intermediate phenotype between allelic disease and MED. Two MED1 patients with an intermediate phenotype showed mild short stature starting in late childhood, while the height SDS values in other MED1 patients ranged from −1.2 to −1.5. Finger joint contractures were prominent in MED4 and MED7. Hip joint involvement was more pronounced in all types except MED2 and MED3, in which knee involvement was more prominent. Radiological examination of the hand revealed decreased ossification and ragged appearance of the carpal bones in MED1, while advanced ossification was present in MED4 and MED7. In early childhood, a typical mini-epiphysis was seen on hip radiographs in MED1, whereas a ‘Swedish key’ appearance was typical in MED7.

## 4. Discussion

The frequency of genes causing MED varies by ethnicity. A study from the European Skeletal Dysplasia Network reported a *COMP* rate of 66%, a *MATN3* rate of 24%, and a *COL9A2/COL9A3* rate of 10%. In contrast, MED cohort studies in Korea and Japan reported lower *COMP* (43% and 37%), and higher *MATN3* (55% and 47%) and *COL9A2/COL9A3* (16%) rates [7,8,24]. Biallelic *SLC26A2* variants have also been reported to account for almost 10–25% of all MED cases [5,25]. In our study, the frequency of pathogenic variants was 47% in *COMP*, 17.6% in *SLC26A2*, and 11.8% each in *MATN3*, *COL9A2*, *COL9A3*, and *CANT1.* The frequency of the affected gene varied between eastern and western countries.

Pseudoachondroplasia and MED1, two allelic diseases, share some clinical and radiographic abnormalities; however, individuals with PSACH are characterized by short-limb dwarfism and SEMD on radiographs [7]. In a study investigating the correlation between genotype and phenotype in 300 patients with *COMP* mutations, pathogenic variants in exons 9–12, which encode the fourth and fifth calcium-binding T3 repeat domains, have mainly been associated with MED1, while variants in exons 13 and 14, encoding the sixth and seventh domains, have been associated with PSACH [26]. There is no clear genotype–phenotype correlation; however, approximately 30% of PSACH patients have the pathogenic variant p.Asp473del [14] and the recurrent pathogenic variant p.Arg718Trp is associated with a mild form of MED1 [27]. Furthermore, many studies have shown that the same variant may result in both PSACH and MED1 [17,18,28,29,30]. We identified pathogenic variants in exons 13 and 14 of *COMP* in three families and in exons 9–11 in five families. One patient presented here had a clinical phenotype consistent with an intermediate phenotype between MED1 and PSACH; the p.Thr529Ala variant in exon 14 has also been identified in a patient with PSACH [16]. This child developed mild short stature after the age of 7 (Table S1). In addition, our patient with a novel p.Asp378Asn variant in exon 10 had an intermediate phenotype between MED1 and PSACH; he had mild short stature and mild spinal and metaphysis irregularities that developed at the age of 9. Our other eight patients had typical MED1 findings. However, the p.Gly440Arg variant in one of these patients has previously been associated with PSACH [17].

Mild to moderate short stature (around or slightly below the third percentile) may appear at 5–6 years of age; however, there are many examples of adults with normal height in MED [3,28]. In our MED1 group, height was normal or at the lower end of the normal range in eight patients at the last examination. Hip joint involvement has been reported to be more frequent in MED1, as observed in our patients [4,28]. Six children had genu valgum, a lower extremity deformity common in MED1, and four underwent knee correction surgery [1,3,28,31]. In addition, we observed pes planus in six out of ten patients. In a study involving ten family members affected by the *COMP* mutation, nine individuals had pes planus [32]. Among our patients aged 10–15 years at the last examination, although they had developed worsening waddling gait, fatigue, difficulty climbing stairs, and pain in the hip and/or knee joints, they had not lost the ability to walk independently. Their radiological features differed from those of other MED types in early childhood, showing irregular, ragged, and delayed ossification of the carpal bones, irregular and oblique acetabula, and small, rounded femoral head epiphyses (mini-epiphyses). These typical findings, along with the ‘glacier crevice sign’, disappeared as the knee epiphyses closed after adolescence. Therefore, it is very difficult to diagnose MED1 radiologically in adulthood [1,3,4,16,28]. In our patients, we observed that after late childhood, only slight irregularity persisted in the carpal bones, the proximal and distal femoral epiphyses became flattened and irregular, the femoral neck became shorter and wider, and coxa vara developed with age.

Monoallelic mutations in *COL9A1-COL9A3* cause MED types 2, 3, and 6, while biallelic mutations cause the Stickler phenotypes [33]. In MED2 and MED3, the knees are more affected than the hips [1,3,9]. Previous studies have shown that monoallelic variants causing MED2 and MED3 are localized only at the splice donor or acceptor site of *COL9A2* and *COL9A3*, resulting in skipping of exon 3 [1,7,12,19,34]. This leads to loss of binding of collagen type IX to matrilin-3 and collagen type II [35]. In our two cases from one family, a c.186+6T>G variant was detected in intron 3 of *COL9A2*, and the clinical features of these patients included a stiff and painful knee joint, while the hip was not affected. Although this variant is classified as a VUS in databases, one study has shown that it leads to the MED2 phenotype [19]. A novel likely pathogenic c.148-2delA variant was detected in intron 2 of *COL9A3* in a child and an affected mother. The child had knee joint involvement with onset at age 11, and the affected mother required knee replacement surgery for osteoarthritis at age 40. One study reported that in cases with a splice variant in intron 2 of *COL9A3*, all affected family members had variable knee pain and limitations [34].

MED4, caused by biallelic pathogenic variants in *SLC26A2*, differs from the allelic disorder, diastrophic dysplasia, by the absence of severe short stature, cleft palate, ’hitchhiker’s thumb’, or cystic external ear. Unlike other MED forms, the MED4 phenotype observed in our patients manifests with clubfoot at birth, joint contractures, moderate brachydactyly, radiologically advanced carpal bone ossification, and a ’double-layered patella’ appearance observed in some patients [2,3,20]. We detected a biallelic c.1957T >A variant in *SLC26A2*, which is the third most common variant in Europe and the most common in Russia, in one patient [20,36,37,38]. In the second family, compound heterozygous VUSs were detected in the two affected siblings; the in-frame deletion (c.1404_1406del) has not been reported, while the other missense VUS (c.2057G>A) has been reported in patients with MED4 [21]. One study reported that patients with the homozygous c.1957T>A variant developed milder forms with later onset [38]. In our study, although the patient with this variant exhibited early-onset findings, disease progression was slow until age 11, with her height remaining within the normal range. The fourth patient was found to have a reported biallelic VUS (c.398C>T) in *SLC26A2* and had an intermediate phenotype between diastrophic dysplasia and MED4, closer to MED, as in the reported patients [39].

Monoallelic variants of *MATN3* result in MED5, characterized by gait abnormalities, irregular epiphyses of the knee and/or hip, and metaphyseal striation [40]. However, patients with biallelic pathogenic variants of *MATN3*, which cause SEMD Borochowitz–Cormier-Daire type, exhibit short-limbed stature and severe genu varum deformity. Clinical signs have not been observed in heterozygous parents [15]. Most pathogenic variants associated with these two phenotypes are missense variants in exon 2 of *MATN3*, which affect von Willebrand factor type A (vWFA)-like domains of the protein and disrupt the folding of matrilin-3 [41]. Interestingly, we identified two biallelic missense VUSs in exon 2 of *MATN3* in two patients, leading to MED5 phenotype. Of our two patients, one had knee involvement and the other had hip dysplasia, both starting in late childhood. The patient with knee involvement had typical metaphyseal striae, while neither patient had involvement of the spine or metaphyseal regions. In their parents, who were heterozygous for these variants, there was no epiphyseal involvement.

Patients with reduced penetrance and intra- and inter-familial variability in MED associated with *MATN3* have been reported [1,8,40,42]. One study described MED5 patients with the p.Arg121Trp mutation exhibiting marked clinical and radiological variability, with epiphyseal abnormalities ranging from mild to severe [40]. Pathogenic variants in *MATN3* cause misfolding and accumulation of the proteins in the rough endoplasmic reticulum (ER) of chondrocytes, resulting in ER stress [11]. Although protein accumulation and ER stress are common pathological mechanisms, Fresquet et al. [43] demonstrated that some α-helical vWFA mutations in exon 2, such as p.Lys231Asn, permit correct folding and secretion of mutant matrilin-3. These studies indicate that some mutations result in an incomplete phenotype and contribute to the heterogeneity in the pathogenesis of MED5. In their comprehensive review of the pathogenesis of MED and MED-like disorders caused by mutations in extracellular matrix genes (*COMP*, *MATN3*, *COL2A1*, and *COL9A1-A3*), Dennis et al. [12] suggested that biallelic variants in this domain of *MATN3* may cause a wide range of clinical conditions, including SEMD, homozygous MED, osteoarthritis, and asymptomatic cases due to decreased penetrance. They also suggested that reporting VUS in these genes, detected during routine genetic screening of MED patients, may clarify some possible conditions. In our study, the finding that biallelic VUS in this domain of *MATN3* in two different families lead to the MED5 phenotype supports this hypothesis.

In this study, two children with disease-related variants in *CANT1* had mild midface hypoplasia, borderline short stature, flexion contractures of the fingers, clubfoot or pes planus, the characteristic ‘Swedish key’ appearance of the proximal femur, and advanced carpal bone age on radiographs. However, neither exhibited severe short stature, short limbs, or multiple joint dislocations attributable to the allelic disorder, Desbuquois dysplasia type 1 [44]. It may be difficult to clinically differentiate Desbuquois dysplasia from MED7 in early childhood. One of our patients, whose molecular diagnosis and initial clinical findings were previously reported as diastrophic dysplasia [23], was followed until the age of 18. At this age, the height SDS had decreased from −2.2 to −3.7. He experienced no joint pain, no difficulty walking or climbing stairs, and his school performance was very good, exhibiting an intermediate phenotype closer to MED7 than Desbuquois dysplasia. Our other patient, aged 3 years and followed for only one year, shared the same pathogenic variant and clinical features as six patients recently reported from Türkiye, who exhibited a phenotype intermediate between Desbuquois dysplasia and MED7 [22].

One limitation of our study is our inability to obtain DNA from the parents in one family carrying biallelic VUS in the *MATN3* gene, which prevented segregation analysis, as well as the lack of functional validation for two variants. However, reporting these observations will help ensure that future research focuses on functional studies to validate novel variants and associated phenotypes, contributing to a better understanding of these complex disease mechanisms.

## 5. Conclusions

The frequency of *COMP* among MED types in this study was 47%, and seven novel variants were identified. We found no complete genotype–phenotype association regarding whether specific *COMP* heterozygous variants would lead to MED1. As an observation supporting a previously reported study, we showed that patients with the homozygous recurrent c.1957T>A variant in *SLC26A2* develop milder forms with later onset. For the first time, biallelic variants in *MATN3* suggested a possible association with MED5 phenotype in two patients. In fifteen children followed between the ages of 11 and 18, a slowly progressing waddling gait and joint pain were present, but walking was not completely restricted, although the effects varied. By comparing radiological findings at different ages, we highlight important information that will help in the clinical diagnosis and prognosis of patients. We believe that the information obtained may contribute to the differential diagnosis and follow-up of this group of diseases, as well as to understanding the pathogenesis of diseases related to bone metabolism.

## Figures and Tables

**Table 1:** The disease-related variants found in the patients with multiple epiphyseal dysplasia.

F/P Number	Associated Phenotype/Gene	Transcript	Zygosity	Variant	Protein Change	Location	Acmg Classification	Reference for Variant
F1/P1	MED1/*COMP*	NM_000095.3	Monoallelic	c.1585A>G	p.Thr529Ala	Exon 14	Pathogenic	[16]
F2/P2	MED1/*COMP*	NM_000095.3	Monoallelic	c.1318G>A	p.Gly440Arg	Exon 13	Pathogenic	[17]
F3/P3	MED1/*COMP*	NM_000095.3	Monoallelic	c.1318_1320 dup	p.Gly440dup	Exon 13	Likely Pathogenic	This study
F4/P4	MED1/*COMP*	NM_000095.3	Monoallelic	c.1189G>C	p.Asp397His	Exon 11	Likely pathogenic	[7]
F5/P5, P6	MED1/*COMP*	NM_000095.3	Monoallelic	c.1186_1188del	p.Lys396del	Exon 11	Pathogenic	This study
F6/P7	MED1/*COMP*	NM_000095.3	Monoallelic	c.1132G>A	p.Asp378Asn	Exon 10	Pathogenic	This study
F7/P8	MED1/*COMP*	NM_000095.3	Monoallelic	c.1043G>A	p.Cys348Tyr	Exon 10	Pathogenic	[7]
F8/P9, P10	MED1/*COMP*	NM_000095.3	Monoallelic	c.905A>T	p.Asp302Val	Exon 9	Pathogenic	[18]
F9/P11, P12	MED2/*COL9A2*	NM_001852.4	Monoallelic	c.186+6T>G	–	Intron 3	VUS (PM2, PP3)	[19]
F10/P13, P14	MED3/*COL9A3*	NM_001853.4	Monoallelic	c.148-2del	–	Intron 3	Likely Pathogenic	This study
F11/P15	MED4/*SLC26A2*	NM_000112.4	Biallelic	c.1957T>A	p.Cys653Ser	Exon 3	Pathogenic	[20]
F12/P16, P17	MED4/*SLC26A2*	NM_000112.4	Biallelic	c.1404_1406del/ c.2057G>A	p.Leu469del/ p.Cys686Tyr	Exon 3/ Exon 3	VUS (PM2, PM4)/ VUS (PM1, PM2, PP3)	This study/[21]
F13/P18	MED4/*SLC26A2*	NM_000112.4	Biallelic	c.398C>T	p.Ala133Val	Exon 2	VUS (PM2, PP3)	[21]
F14/P19	MED5/*MATN3*	NM_002381.5	Biallelic	c.271T>G	p.Ser91Ala	Exon 2	VUS (PM2, PP3)	This study
F15/P20	MED5/*MATN3*	NM_002381.5	Biallelic	c.523A>C	p.Thr175Pro	Exon 2	VUS (PM2, PP3, PM1)	This study
F16/P21	MED7/*CANT1*	NM_001159773.2	Biallelic	c.375G>C	p.Trp125Cys	Exon 2	Likely pathogenic (PM2, PP3, PS3)	[22]
F17/P22	MED7/*CANT1*	NM_001159773.2	Biallelic	c.-342+1G>A	–	Intron 1	VUS (PM2, PP3, PP5)	[23]

MED: Multiple epiphyseal dysplasia; VUS: variant of unknown significance; F: family; P: patient.

**Table 2:** Comparison of clinical and radiological features of patients with multiple epiphyseal dysplasia.

Disease-Related Gene/ MED Phenotype	Monoallelic Variants			Biallelic Variants			Total
	*COMP* */MED1* *n* = 10	*COL9A2* */MED2* *n* = 2	*COL9A3* */MED3* *n* = 2	*SLC26A2* */MED4* *n* = 4	*MATN3* */MED5* *n* = 2	*CANT1* */MED7* *n* = 2	*n* = 22
**Onset age of initial sign (years)** 0–5 6–10 >11 NA	7/10 1/10 – 2/10	1/2 1/2 – –	– 2/2 – –	4/4 – – –	– – 2/2 –	2/2 – – –	14/22 4/22 2/22 2/22
**Initial Sign** Waddling gait Joint pain Club feet	5/10 5/10 –	– 2/2 –	– 2/2 –	2/4 1/4 1/4	– 2/2 –	– – 1/2	7/22 12/22 2/22
**Mean height SDS** 1–3 years 4–7 years 10–15 years Final	– −1.3 (5/10) −1.5 (7/10) −1.5 (2/10)	– – −0.4 (1/2) −1.5 (1/2)	– – −0.9 (1/2) −1.9 (1/2)	−2 (3/4) −2.2 (2/4) −1.7 (2/4) –	– – −1.2 (2/2) –	−2.7 (2/2) – – −3.7 (1/2)	(−2.3) 5/22 (−1.7) 7/22 (−2.2) 13/22 (−2.1) 5/22
**Clinical features**							
**Waddling gait**	8/10	1/2	2/2	3/4	2/2	0/2	15/22
**Difficulty in climbing stairs**	7/10	1/2	2/2	1/4	1/2	1/2	13/22
**Joint pain** Hip Knee	9/10 4/10	– 2/2	– 2/2	2/4 –	1/2 1/2	– –	12/22 9/22
**Fatigue after exercise**	8/10	2/2	2/2	2/4	2/2	0/2	16/22
**Genu valgum** **Genu varum**	4/10 1/10	– –	– 1/2	– –	1/2 –	– 1/2	5/22 3/22
**Scoliosis**	–	–	–	1/4	–	1/2	2/22
**Limited joint** Elbow Finger Knee	8/10 – –	– – –	2/2 – 2/2	3/4 4/4 –	– – –	2/2 2/2 –	15/22 6/22 2/22
**Brachydactyly**	1/10	–	1/2	3/4	–	1/2	6/22
**Pes planus**	6/10	–	–	1/4	–	1/2	8/22
**Club feet**	–	–	–	1/4	–	1/2	2/22
**Most involved joint** Hip Knee	8/10 2/10	– 2/2	– 2/2	4/4 –	1/2 1/2	2/2 –	15/22 7/22
**Surgery** Hip osteoarthritis Genu valgum Club feet	2/10 2/10 –	– – –	– 2/2 –	1/4 – 1/4	– 1/2 –	– – 1/2	3/22 5/22 2/22
**Radiological features**							
**Hand** Ragged carpal bones Irregular and small carpal bones Short metacarpals/phalanges Advanced carpal ossification	7/10 8/10 4/10 –	1/2 2/2 – –	– 2/2 1/2 –	– 4/4 3/4 4/4	– 2/2 – –	– – – 2/2	8/22 18/22 8/22 6/22
**Hip** Irregular/flat acetabular roof Small-round femoral head Broad, short femoral neck Coxa valga Coxa vara Swedish key appearance	6/10 8/10 10/10 4/10 1/10 –	1/2 – 1/2 – – –	– 2/2 1/2 1/2 –- –-	3/4 4/4 4/4 – 4/4 –	– 2/2 2/2 – 1/2 –	2/2 2/2 2/2 2/2 – 2/2	12/22 18/22 20/22 7/22 6/22 2/22
**Knee** Irregular/small/flat epiphyses Double-layered patella	9/10 –	2/2 –	2/2 –	2/4 1/4	1/2 –	2/2 –	18/22 1/22
**Mild metaphyseal irregularity**	3/10	–	1/2	–	1/2	–	5/22
**Mild platyspondyly**	3/10	–	–	4/4	–	1/2	9/22

MED: Multiple epiphyseal dysplasia; NA: Not available; SDS: Standard deviation score.

## Data Availability

The data that support the findings of this study are available from the corresponding author upon reasonable request.

## Supplementary Materials

The following supporting information can be downloaded at: https://www.mdpi.com/article/10.3390/genes17040463/s1, Table S1: Clinical and radiological features of the patients with multiple epiphyseal dysplasia type 1; Table S2: Clinical and radiological features of the patients with multiple epiphyseal dysplasia type 2–5 and 7.

## Abbreviations

The following abbreviations are used in this manuscript:

MED	Multiple epiphyseal dysplasia
SDS	Standard deviation score
VUS	Variants of uncertain significance
PSACH	Pseudoachondroplasia
SEMD	Spondyloepimetaphyseal dysplasia
ER	Endoplasmic reticulum
