# Supplementary material for: Similarities and Differences of Multiple Epiphyseal Dysplasias: Genetic Features and Natural Course in 22 Patients
Source: Genes (Basel). 2026 Apr 15;17(4):463. doi: 10.3390/genes17040463 (PMC13115595; doi:10.3390/genes17040463)
Supplement: Supplementary file 1 [file genes-17-00463-s001.zip › genes-4243502-supplementary.pdf]

Table S1: Clinical and radiological features of the patients with multiple epiphyseal dysplasia type 1**.**

**Clinical diagnosis /Gene**	**MED1/*COMP***									
**Family Number**	**1**	**2**	**3**	**4**	**5**		**6**	**7**	**8**	
**Patient Number**	**1**	**2**	**3**	**4**	**5**	**6**	**7**	**8**	**9**	**10**
**Gender(F/M)**	F	F	F	F	M	F	M	M	M	M
**Parental consanguinity/ Familial history**	-/-	-/-	-/-	-/-	-/+		-/-	+/-	-/+	
**Age (initial/last visit) year**	5.1/9.5	6.3/10.4	7,8/14.2	10.8/10.8	13/14	35/35	5.7/15	9.5/15	6.6/14	39/39
**Height SDS (initial/last visit)**	-1.8/-3.2	-1.3/-1.3	-0.7/-1.3	-1.6/-1.6	+2/+2	-0.36/-0.36	-1.5/-2.9	-1,4/-1.7	-0.5/-1.5	-2.1/-2.1
**Initial sign**	Fatigue and pain after exercise	Waddling gait	Waddling gait	Hip pain after exercise	Hip and Knee pain	Hip pain	Waddling gait	Waddling gait	Waddling gait	Hip pain
**Age of initial sign (year)**	2	5	5	5	10	NA	2	2	4	NA
**Clinical features**										
Waddling gait	+	+	+	+	-	-	+	+	+	+
Difficulty in climbing stairs	+	+	+	-	-	-	+	+	+	+
Joint pain (Hip/Knee)	+/-	+/+	-/-	+/-	+/+	+/-	+/+	+/-	+/-	+/+
Fatigue to long distance walking	+	+	+	+	-	+	+	-	+	+
Genu varum/valgum	-/+	+/-	-/+	-/+	-/-	-/-	-/+	-/-	-/-	-/-
Brachydactyly	-	+	-	-	-	-	+	-	-	-
Joint stiffness	elbow	elbow	elbow	elbow	-	-	elbow	elbow	elbow	elbow
Finger/knee joint hypermobility	+/+	+/-	+/+	-/+	-/+	-/-	+/+	+/-	-/-	-/-
Pes planus	+	+	+	+	-	-	+	-	+	+
Surgery	genu valgum	-	genu valgum	-	-	-	-	-	-	hip osteoarthritis
**Radiological features**										
Ragged/ small/ irregular carpal bones	+/+/+	+/+/+	+/+/+	-/-/+	-/-/+	-/-/-	+/+/+	+/+/+	+/+/+	-/-/-
Short metacarpals/phalanges	+/+	+/-	+/-	-/-	-/-	-/-	+/-	+/-	-/-	-/-
Irregular acetabular roof	+	+	+	+	+	+	+	+	+	+
Irregular/flattening/ small-round femoral head	-/-/+	-/-/+	-/-/+	+/+/-	-/+/+	-/-/+	-/-/+	-/-/+	-/+/-	+/+/-
Broad/short femoral neck	+/-	+/-	+/-	+/-	+/-	-/+	+/-	+/-	+/-	+/-
Coxa vara/Coxa valga	-/+	-/+	-/+	-/-	+/-	-/-	-/+	-/-	-/-	-/-
Irregular/small knee epiphyses	+/-	-/+	+/+	+/+	+/-	-/-	+/+	+/-	+/-	-/-
Metaphyseal irregularity	+	-	+	-	-	-	+	-	-	-
Mild platyspondyly or irregular vertebral endplates	+	+	-	-	-	-	+	-	-	-
**Most involved joint**										
Hip	+	+	+	++	+	+	++	++	++	++
Knee	+	++	+	+	+	+	+	+	+	+

MED: Multiple epiphyseal dysplasia; F: female; M: male; SDS: Standard deviation score; +: Mild-Moderate involvement; ++: Severe involvement

Table S2: Clinical and radiological features of the patients with multiple epiphyseal dysplasia type 2-5 and 7.

Clinical phenotype / Gene	MED2/*COL9A2*		MED3/*COL9A3*		MED4/*SLC26A2*				MED5/*MATN3*		MED7/*CANT1*	
**Family Number**	9		10		11	12		13	14	15	16	17
**Patient Number**	11	12	13	14	15	16	17	18	19	20	21	22
**Gender(F/M)**	M	M	F	F	F	M	F	M	M	M	M	M
**Parental consanguinity/ Familial history**	-/+		-/+		+/-	+/+		Same village /-	-/-	-/-	+/-	+/-
**Age (initial/last visit) year**	10/14	45/45	12.3/21	49/49	3.5/11.5	7.4/10.4	3.5/6.5	2.5/3.5	14/16.5	14/17	3/4	2.5/18
**Height SDS (initial/last visit)**	-0.4/-0.35	-1.5/-1.5	-0.91/-1.4	-2.6/-2.6	0.1/-1.5	-2.2/-1.9	-2.3/-2.2	-2.7/-2.7	-3.2/-3.2	+2/+2	-3.2/-3.2	-2.4/-3.7
**Initial sign**	Knee pain	Knee pain	Knee pain	Knee pain	Waddling gait	Waddling gait	Waddling gait	Club feet	Knee pain	Hip pain	Pes planus	Club feet
**Age of initial sign (year)**	4	10	5	6	2	5	2.5	newborn	12	13	1	newborn
**Clinical features**												
Waddling gait	+	-	+	+	+	+	+	-	+	+	-	-
Difficulty in climbing stairs	+	-	+	+	+	-	-	-	+	-	-	-
Joint pain	Knee	Knee	Knee	Knee	Hip	Hip	-	-	Knee	Hip	-	-
Fatigue to long distance walking	+	+	+	+	-	+	+	-	+	+	-	-
Genu varum/valgum	-	-	+/-	-/-	-/-	-/-	-/-	-/-	-/+	-/-	-/-	+/-
Brachydactyly	-	-	-	+	+	+	+	+	-	-	-	-
Limited joint	-	-	Elbow	Elbow	Finger	Elbow, finger, hip	Elbow/finger	Elbow/finger	Knee	-	Elbow	Elbow
Joint hypermobility (Finger/knee)	-	-	-	-	-	-	-	-	-	-	-	-/+
Early osteoarthritis/Surgery	-/-	-/-	-/Knee	+/Knee	-/Hip	-/-	-/-	-/Club feet	-/Knee	-/-	-/-	-/Club feet
Club feet /pes planus	-	-	-	-	-	-	-	+/-	-	-	-/+	+/-
Other	-	-	-	-	-	-	-	-	-	-	-	MVP
**Radiological features**												
Ragged/small/irregular carpal bones	+/+/+	-/+/+	-/+/+	-/-/+	-/+/+	-/-/+	-/-/+	-/-/+	-/+/+	-/+/+	-/-/-	-/-/-
Advanced carpal ossification	-	-	-	-	+	+	+	+	-	-	+	+
Short metacarpal/ metatarsal bones	-/-	-/-	-/-	+/-	-/+	+/+	+/+	+/+	-/-	-/-	-/-	-/-
Irregular/flattening acetabular roof	+/-	-/-	-/-	-/-	+/-	+/-	+/-	-/-	-/-	-/-	-/+	-/+
Irregular/flattening/hypoplastic femoral head	-/-/-	-/-/-	-/+/-	-/+/-	-/+/-	-/+/-	-/+/-	-/+/-	-/+/-	+/+/-	-/+/-	-/+/-
Short/broad femoral neck	-/-	+/-	-/-	+/-	+/+	+/+	+/+	+/+	+/-	+/-	+/+	+/+
Swedish key appearance	-	-	-	-	-	-	-	-	-	-	+	+
Coxa vara/valga	-/-	-/-	-/+	-/-	+/-	+/-	+/-	+/-	+/-	-/-	-/+	-/+
Irregular/flattening/ small knee epiphyses	+/+/+	+/-/-	+/+/+	+/-/-	-/-/-	-/+/-	-/+/-	-/-/-	+/+/-	-/-/-	+/-/-	+/-/-
Double layered patella	-	-	-	-	+	-	-	-	-	-	-	-

Metaphyseal irregularity<br>Metaphyseal stria	-<br>-	-<br>-	-<br>-	+<br>-	-<br>-	-<br>-	-<br>-	-<br>-	+<br>+	-<br>-	-<br>-	-<br>-
Mild platyspondyly or irregular vertebral endplates	-	-	-	-	-	+	+	+	-	-	+	+
**Most involved joint**<br>Hip<br>Knee<br>Hand	 -<br>++<br>+	 -<br>+<br>-	 -<br>++<br>+	 +<br>++<br>+	 ++<br>-<br>-	 ++<br>-<br>-	 ++<br>-<br>-	 +<br>-	 -<br>++<br>-	 ++<br>-<br>-	 +<br>+<br>+	 +<br>+<br>+
**Other**	-	-	-	-	-	-	-	Scoliosis	-	-	Osteopenia	Osteopenia, phalangeal dislocation

MED: Multiple epiphyseal dysplasia; F: female; M: male; SDS: Standard deviation score; +: Mild-moderate involvement; ++: Severe involvement; MVP: Mitral valve prolapse
